# Neurophysiological Processing of an Emotional Task is Sensitive to Time-of-Day

**DOI:** 10.5334/jcr.148

**Published:** 2017-05-23

**Authors:** Isaac Chayo, Mercedes Fernandez, Samantha Sandor, Jaime L. Tartar

**Affiliations:** 1 Department of Psychology and Neuroscience, Nova Southeastern University, Ft. Lauderdale, Florida, US

**Keywords:** Attention, Circadian, Emotion, ERP, IAPS, LPP

## Abstract

Previous work from our laboratory has shown that a measure of attention to emotionally-charged stimuli, the late positive potential (LPP) event related potential (ERP), distinguished neutral from emotional pictures on a baseline day, but not after sleep deprivation. Here we sought to extend these findings and address the uncertainty about the effect of time-of-day on emotion processing by testing a morning group (8:00–10:00 a.m., n = 30) and an evening group (8:00–10:00 p.m., n = 30). We also examined the extent of diurnal changes in cortisol related to the emotion processing task. Results from this study mirrored those found after one night of sleep deprivation. Compared to the morning group, the LPP generated by the evening group (who had a greater homeostatic sleep drive) did not distinguish neutral from emotionally-charged stimuli. New to this study, we also found that there was a time-of-day effect on positive, but not negative pictures. While, as expected, cortisol levels were higher in the morning relative to the evening group, there was no relationship between cortisol and the LPP ERP emotion measure. In addition, neither time-of-day preference nor sleep quality was related to the LPP measure. These findings show that, similar to what occurs after sleep deprivation, increased sleep pressure throughout the day interferes with attention processing to emotional stimuli.

## Introduction

General neurocognitive deficits with increased time awake have been well-documented in the literature [1]. For example, increased time awake results in decreased performance and delayed reaction times on a psychomotor vigilance task [2,3] and impairs cognitive performance and subjective alertness [4]. However, few studies have evaluated the specific consequences of increased homeostatic sleep drive on emotion processing and, to our knowledge, no previous study has determined whether time-of-day has an impact on neurophysiological processing of emotional stimuli. Nevertheless, this effect is likely since subjective measures suggest that well-being [5] and emotional reactivity [6] are impaired with increased time awake. Moreover, psychophysiological studies have shown that event-related potentials (ERP) components are affected by time of day, by morning vs. evening activity preferences, and by other factors that change with time of day [7,8]. For instance, one study investigating time of day, morning versus evening activity preferences, and food intake on the P300 component of the ERP revealed that when tested in the morning, evening preferring participants who had not consumed food within 3 hours of testing showed relatively smaller P300 amplitudes relative to those tested in the evening; morning preferring participants who had not eaten within 3 hours of testing showed relatively smaller P300 when tested in the evening compared to those tested in the morning [9]. The authors concluded that their findings may be due to differences in arousal level, and arousal level likely influences processing of emotional stimuli. Although this study did not examine cortisol levels, it is also possible that diurnal fluctuation in cortisol levels are related to the time-of-day effects observed in these studies. Cortisol release is entrained to circadian patterns with peak concentrations in the early morning and it reaches its nadir in the evening. Exogenous synthetic cortisol administration has been shown to increase negative affect and arousal ratings for unpleasant pictures in a picture rating task [10] and to increase response time to emotional words in an affective Go/No-go task [11]. In addition, our previous research shows that acute stress induction enhances the LPP ERP response to an emotional picture rating task after 30 minutes- a time when cortisol levels were at their highest [12].

Like the P300, the LPP component of the ERP is specifically established as a sensitive measure of attention to emotionally-charged stimuli- especially emotional stimuli of negative valence [13,14,15]. Induction of the LPP is thought to serve as a neurobiological correlate of motivated attention to stimuli of adaptive significance (e.g., sex, death). In other words, because these stimuli are inherently arousing, they require the preferential allocation of limited attention resources [16].

Given that the P300 induction during an emotion task has been shown to be sensitive to time-of-day [7,9] and that the P300 and the LPP share overlapping time windows and processing characteristics [17], it is plausible that as with the P300, the LPP will also be sensitive to time-of-day. In addition, our previous study showed that the LPP ERP is vulnerable to increased time awake; we explored the impact of 24-hours of sleep deprivation on neurophysiological measures of emotion processing through the LPP and found disruptions in normal emotion-state switching. Specifically, the neutral and emotional pictures elicited similar LPP in sleep deprived participants. Thus, after one night of sleep deprivation, subjects were unable to allocate the attentional resources necessary to discriminate between neutral and emotional pictures [18].

To replicate and extend our previous findings on emotion changes after sleep deprivation, the current study aimed to assess the impact of increased homeostatic sleep drive as a function of time of day on emotion picture processing. Based on previous work [7,9,10,11], we also examined the extent to which morningness and eveningness preferences and diurnal fluctuations in cortisol related to potential LPP differences in the morning vs. evening. We hypothesized that the LPP effects we observed after sleep deprivation could be extended to time-of-day; that increased sleepiness throughout the day would result in emotional instability in the LPP measure. This hypothesis is supported by our previous finding that sleep deprivation impairs the ability to switch attention between emotional and non-emotional pictures [18] and is also consistent with previous work that showed impairment in the ability to shift non-emotional attention between tasks following sleep deprivation [19,20,21,22]. We specifically predicted that in response to a succession of randomly and rapidly presented emotional and non-emotional pictures, the LPP amplitude to the neutral pictures would be increased and not significantly different from the LPP to the emotional pictures in the evening relative to the morning condition.

## Methods and materials

### Participants

Right-handed participants with normal or corrected-to-normal vision and without sleep or psychological disorders participated in this study. Participants were recruited through the University’s research participation website and were assigned to either an 8:00–10:00 a.m. morning testing condition (n = 30, Mean Age = 22, SD = 5.23; 18 females) or an 8:00–10:00 p.m. evening testing condition (n = 30, Mean Age = 19, SD = 2.43; 16 females). This study was approved by the Institutional Review Board at Nova Southeastern University, and all participants were compensated for their time with research credit or a gift card.

### Questionnaires

*Screening Questionnaire* – This brief interview was conducted to determine eligibility to participate in the study. Students interested in participating were asked about their handedness, vision, and history of psychological and sleep disorders. Only those who met eligibility criteria were scheduled for testing in the psychophysiology laboratory.

*Demographics Questionnaire* – This self-report measure was used to obtain age, sex, and race of participants.

*Morningness-eveningness questionnaire (MEQ)* – We determined a possible influence of chronotype using the MEQ scores [23]. This instrument contains 19 items which tap an individual’s waking and bed times, preferred times for engaging in a variety of activities, and subjective alertness at sleep and wake times. Internal consistency is good, with a Cronbach α = 0.82 [24]. Test-retest reliability correlations across a number of studies have been reported to range between 0.84 and 0.95, and the instrument has been validated against a variety of circadian-related variables such as body temperature, melatonin and cortisol secretion, and sleep habits [25].

*Pittsburg Sleep Quality Index (PSQI)* – Sleep quality was measured using the self-rated PSQI questionnaire [26]. The PSQI is a 19-item instrument of sleep quality. With strong psychometric properties, the instrument is used widely. The instrument exhibits high internal consistency (Cronbach α = 0.83), adequate test-retest reliability scores across the sleep-related components of the instrument for a nonclinical sample (from 0.44 to 0.70), and the ability to differentiate controls from sleep-disordered groups [26].

### Stimuli

Pictures from the International Affective Picture System (IAPS) [16] were used to elicit ERPs. The IAPS normative ratings were used to select a total of 105 pictures, 35 from each valence (positive, negative, neutral) category.

### Cortisol

Human enzyme immunoassay (EIA) kits (Salimetrics LLC, USA) were used to quantify salivary cortisol levels. A saliva sample was collected before EEG recording by unstimulated passive drool. The sample tubes were stored in a –20°C freezer immediately after collection.

### EEG recording

The ongoing EEG was recorded and amplified using Psylab EEG equipment (Contact Precision Instruments, Cambridge, MA) and a cap (Electrocap International, Eaton, OH) fitted with tin cup electrodes (Fz, Cz, Pz, C3, C4, O1, O2) and referenced to linked earlobes. Eye movements were recoded with electrodes placed above and on the outer canthus of the right eye. Electrode impedance was maintained at less than 5 kΩ. The EEG amplifier was set at a gain of 30,000 and the EEG was sampled at 500 Hz. High and low pass filters were set to .1 and 40 Hz, respectively, and a 60 Hz notch filter was active.

EEG data were analyzed with Psylab8 software (Contact Precision Instruments, Cambridge, MA). EEG activity of 1000 ms, including 100 ms prestimulus baseline, was averaged to extract the LPP ERP. The LPP was defined as the average voltage in the 700–900 ms time window after stimulus onset at Cz and Pz electrode locations. This time range was chosen based on the spatial-temporal pattern of maximal LPP generation within this window. We did not analyze the LPP after 1000 ms due to an observed high amount of noise and eye artifact after this period and an apparent N2 component overlapping the earlier time window.

### Procedure and design

Eligible participants were scheduled for testing and instructed not to eat 1 hour prior to their scheduled session. At the psychophysiology lab, participants read and signed a consent form and then completed paper and pencil questionnaires. Saliva samples were taken prior to the start of EEG recording.

The EEG procedure was carried out as described in [18] and was designed to evoke an LPP through the use of a balanced number of emotional and non-emotional pictures. The electrode cap and eye electrodes were affixed and participants sat in front of a computer monitor used to present the pictures. Picture presentation and timing were controlled with Presentation software (Neurobehavioral Systems, LLC). There were 105 trials and each trial began with a 400 ms negative, positive, or neutral picture presented in a pseudorandom order. Following picture presentation, a black screen was presented for the remainder of the trial (4000 ms) during which time participants rated the picture valence by pressing a keyboard button. A white fixation point was present in the center of the screen throughout the experiment.

### Statistical analyses

A Mixed Model Analysis of Variance (ANOVA) was used to examine the effect of picture category and group on the visual LPP amplitude. Group status (morning vs. evening) served as a between-subjects factor and picture category (positive, negative, neutral) served as the within-subject factors. The relationship between the LPP amplitude and sleep questionnaires (MEQ and PSQI) and cortisol was investigated using Pearson’s r. Finally, we confirmed circadian variation in cortisol through an independent samples *t* tests. Post-hoc pairwise comparisons were carried out using a Bonferroni correction for multiple comparisons and independent samples t-tests were used for all planned comparisons. In instances where the sphericity assumption was not met, the reported p-values associated with the F statistics were adjusted via the Greenhouse–Geisser correction. We also tested for possible correlations between cortisol and the LPP measure as well as possible correlations between the sleep measures (MEQ or the PSQI) and the LPP measure. All reported p values are two-tailed with an a priori significance level of p < 0.05. All statistical analyses were conducted using SPSS (SPSS Inc., IBM).

## Results

### LPP amplitude

**Figure 1:**
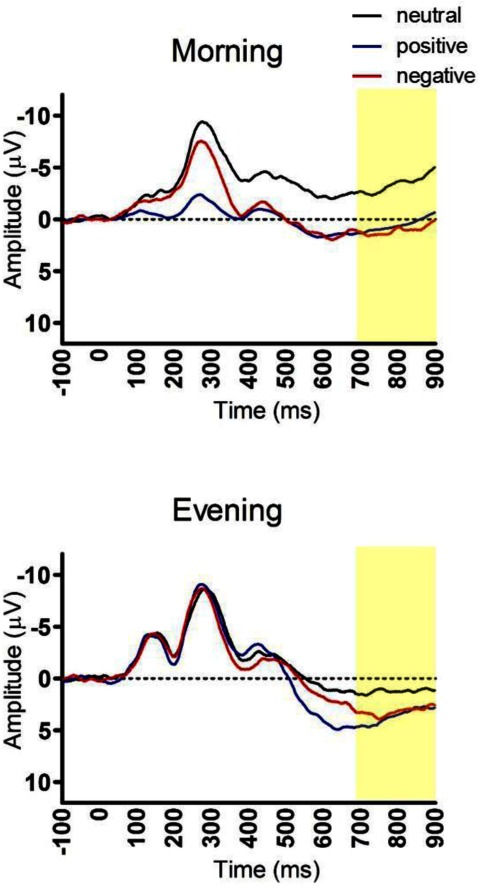
Visual LPP ERPs for morning compared to evening conditions. Participants were exposed to an emotionally positive, neutral, or negative pictures for 400 ms. The average Cz/Pz LPP amplitude (latency range = 700–900 ms) in the evening group was larger than the morning group, and this difference was significant for the neutral and positive picture conditions. Y axis represents voltage (µV) and x axis represents time (ms).

Figure 1 presents the grand average LPPs to the three picture categories (neutral, positive and negative) separated by time-of-day and LPP amplitudes can be found in Table 1. The ANOVA comparing the morning and evening group showed a significant group main effect (F(1,58) = 6.52, partial eta² = 0.10, p = 0.01). Bonferroni comparisons showed that the morning group had a smaller LPP (mean = –1.47, SE = 1.10) relative to the evening group (mean = 2.49, SE = 1.10). There was also a significant main effect for picture (F(2,116) < 0.07, partial eta² = 0.25, p < 0.01). Bonferroni comparisons showed that, relative to neutral pictures (mean = –1.72 SE = 0.80), the LPP was significantly larger for positive (mean = 1.47 SE = 0.93, p < 0.01) and negative pictures (mean = 1.78 SE = 0.81, p < 0.01). There was not a significant picture × group interaction (F(2,116) = 1.49, p = 0.23). We carried out planned comparisons that were made based on our previous findings and current hypothesis [27]. These analyses showed that increased sleep pressure throughout the day alters emotion processing. The between group post hoc t-test analyses showed that the LPP amplitude to the neutral pictures was significantly larger in the evening compared to the morning group (t(58) = –2.95, p < 0.01). We further found that the LPP amplitude to the positive pictures was significantly larger in the evening compared to the morning group (t(58) = –2.928, p = .03).

**Table 1:** LPP amplitudes.

Picture Category	Morning Group		Evening Group	

	Mean	SD	Mean	SD

Positive Picture LPP (µV)	–0.63	8.36	3.57*	5.74
Neutral Picture LPP (µV)	–4.17	6.87	0.73**	5.96
Negative Picture LPP (µV)	0.39	6.72	3.17	5.70

Planned comparisons were carried out via independent samples t tests in order to test for differences between the morning and evening groups. These analyses showed that the LPP ERP was significantly larger in the evening group relative to the morning group for the positive and neutral pictures. Asterisks indicate significantly different from the morning group (* = p < 0.05, ** = p < 0.05).

### Picture Ratings

Unlike the LPP ERP measure, time-of-day did not influence picture ratings. The There was not a picture rating × session interaction (F(2,116) = 1.93, partial eta² = 0.03, p = 1.58). As expected, however, there was a significant main effect for picture rating (F(2,116) = 489.12, partial eta² = 0.89, p < 0.01), indicating that the participants were attending to the task. There was not an effect of time-of-day or picture rating on the reaction times (all p’s > 0.05). Table 2 shows the means and standard deviations for picture ratings and reaction times.

**Table 2:** Picture Ratings and Reaction Times.

Picture Category	Morning Group		Evening Group	

	Mean	SD	Mean	SD

Positive Picture Rating	7.05^b,c^	1.21	7.56^b,c^	.97
Positive Picture Reaction Time (ms)	356.69	52.99	357.95	39.15
Neutral Picture Rating	5.19^a,c^	0.55	5.32^a,c^	0.54
Neutral Picture Reaction Time (ms)	359.67	25.61	356.82	36.14
Negative Picture Rating	2.25^a,b^	0.88	2.11^a,b^	0.91
Negative Picture Reaction Time (ms)	358.77	39.60	356.27	28.67

There was a significant main effect for picture ratings F(2,166) = 489.12, p < 0.01, There was not a main effect of time of day on picture ratings or a significant picture rating × time of day interaction. Superscripts indicate significant differences across picture ratings separately for the morning and evening groups. There were no significant main or interaction effects for the reaction time ratings. ^a^ = significantly different from positive picture ratings. ^b^ = significantly different from neutral picture ratings. ^c^ = significantly different from negative picture ratings (all p’s < 0.01).

### Sleep Questionnaires and Cortisol

As expected, cortisol levels were higher in the morning (mean = 0.40, SE = 0.06) relative to the evening (mean = 0.14, SE = 0.01) group (t(58) = 4.00, p < 0.01). The PSQI, MEQ, and cortisol did not correlate with the LPP measures for the neutral, negative, or positive pictures (all p’s > 0.05). In addition, there was not a significant difference on the MEQ (p = 0.49) and PSQI (p = 0.59) between the two groups. The self-reported average hours of sleep on the PSQI was also not significantly different between group (p = 0.98). Mean values for the sleep behavior questionnaires and cortisol levels can be seen in Table 3.

**Table 3:** Sleep Questionnaires and Cortisol.

Picture Category	Morning Group		Evening Group	

	Mean	SD	Mean	SD

PSQI	7.48	3.58	6.90	.2.7
Ave Hours Sleep	6.72	1.41	6.71	2.48
MEQ	44.00	8.17	45.31	10.34
Cortisol (µg/dl)	0.40	0.35	0.14	0.08**

Table 3 shows the results for likely confounding or modifying variables. There was not a significant difference in sleep quality (PSQI) or time-of-day preference (MEQ) between groups, indicating that the LPP changes were not likely related to these factors. Follow up correlations between these sleep measures and the LPP for all pictures categories were also not significant. As expected, there was a time-of day effect of cortisol, but cortisol levels did not relate to the LPP measures for any picture category. ** indicates significantly different from the morning group.

## Discussion

The present results support our previous findings that sleep deprivation increases the LPP amplitude to emotionally neutral pictures [18]. Here, we extend these findings by showing that increased homeostatic sleep pressure throughout the day mimics these effects. Specifically, compared to a morning test group, the LPP amplitude to emotionally neutral pictures was significantly larger during nighttime testing. In other words, participants responded to the neutral pictures as if they were emotional pictures. Unlike our previous findings, however, we also showed that the LPP amplitude for emotionally positive pictures was significantly larger in the evening compared to the morning group. This difference possibly results from the fact that the current study benefited from a larger sample size, thus increasing the statistical power to detect this effect. Nevertheless, the independent and combined results of this and our previous study support the notion that increased time awake causes a disruption to emotion-state switching wherein participants have difficulty switching attention between emotional and non-emotional pictures categories.

Our findings that the LPP ERP amplitude changes with time-of-day is consistent with previous findings that the P300 amplitude is affected by time-of-day [7,8]. However, unlike these previous findings, we did not look at differences in food intake. Since the current study assessed salivary cortisol levels all participants were told not to eat at least 1 hour before testing.

We previously found that the LPP no longer discriminates between emotional and non-emotional picture categories when cortisol levels are highest after the socially evaluated cold-pressor test (SECPT) acute stressor (30 minutes post-stress) [12]. However, here we found that while cortisol levels were significantly higher in the morning group, relative to the evening group, there was no association between this circadian fluctuation in cortisol and the LPP. It is possible that this difference is due to the concomitant limbic activation that is engaged with acute stress, but not the normal circadian fluctuations in cortisol [28]. For example, high cortisol levels with acute stress activates the low-affinity glucocorticoid receptors in the amygdala and hippocampus [29]. Also, the social evaluative component of the SECPT is considered processive stress and as such requires cognitive processing that is relayed primarily through limbic forebrain inputs to the hypothalamus [30]. We also did not detect a relationship between morning vs. evening preferences (MEQ) in time-of-day sensitivity of the LPP ERP. In addition to time-of-day preference, we also assessed sleep quality (PSQI) in order to ensure that any observed LPP changes were not related to sleep behavior since poor sleep quality could explain altered emotion processing [31,32]. However, this did not appear to be the case as there were no group differences in these measures, and there was no correlation between the LPP and either MEQ or PSQI scores. In general, the P300 findings from previous work and our findings suggest that time-of day-effects on the ERP may be due to differences in arousal level as a function of sleepiness accumulated throughout the day, which can influence the neural processing of emotional stimuli. In particular, the findings reported here and after sleep deprivation [18], support the concept that increased time awake leads to increased emotional instability [33] and that one function of sleep is to modulate and regulate affective processing [34].

The fact that participants were able to correctly rate the affective valence of the pictures, in spite of the altered neural processing, supports the idea that increased sleepiness leads to increased emotional instability- wherein neural processing becomes uncoupled from overt behavior. The behavioral picture ratings (1–9, unpleasant– pleasant) were not different between the morning and the evening group, and the ratings also agreed with the expected normative ranges for the IAPS pictures [16]. Thus, despite the changes in the LPP that occur as a function of time-of-day, the emotional and non-emotional stimuli are still correctly classified along their valence dimensions. The extensive activation of subcortical limbic structures involved in LPP generation [35] possibly underlies the uncoupling of the neural measure from the overt behavior measure. The relatively automatic process of LPP generation might reflect subversive changes in emotion processing while overt behavioral response to stimuli can still be recognized as emotional or non-emotional. This is similar to previous work that demonstrated increased sensitivity of the ERP over behavioral measures in detecting clinical impairments in HIV [36] and cognitive changes with bilingualism [37]. Similar to the picture ratings, we did not observe an effect of time-of-day on reaction times. It is possible that the picture rating task in this study was not challenging enough to observe reaction time differences between the groups. Indeed, previous studies that found a time-of-day effect on reaction time used relatively complex tasks such as the serial reaction time task and learning motor sequences [38,39].

One limitation to the study is that because the participants were told not to eat at least 1 hour before arriving for testing, it is possible that depleted blood glucose might have decreased the ability of the participants attend to the task and influenced emotion regulation [40]. However, since both groups were given the same instructions we do not think the fasting state would have influenced our group differences in the LPP ERP measure. Another important consideration in our study results are the parameters used in our experimental design. The picture duration was relatively fast (400 ms) in our study compared to more characteristic longer picture durations of (i.e 1500–6000 ms) [14,41]. In addition, the order of the picture category was randomized, and thus, unpredictable to the participants. Accordingly, impairments in emotion attention switching are conceivably best captured by rapid and unpredictable picture presentation. An additional limitation in the current study is that we cannot be certain if the LPP ERP effects were due to strict circadian factors or to mental fatigue that incurred as a result of exerting-mental-effort throughout the day. However, we were able to rule out the effects of sleepiness and time-of-day preferences in our study through the use of the PSQI and MEQ surveys, respectively.

Overall, the results show that increased sleep pressure throughout the day can interfere with attention processing to emotional stimuli. Importantly, differences between the morning and evening group are not likely to be due to differences in sleep quality or preferred time-of-day to function since there was no difference between the two groups on these measures. These findings suggest that time-of-day should be controlled in studies on ERP LPP response to emotional pictures. We are currently planning a series of investigations to test how the timing and predictability of emotional stimuli are affected by sleep loss. This will allow us to show how different types of emotional experiences can be differentially impacted by sleep loss and also potentially explain variation in the literature on the impact of sleep loss on emotion processing.
